# Supplementary figures and images for: Human cytomegalovirus infection coopts chromatin organization to diminish TEAD1 transcription factor activity
Source: eLife. 2025 Sep 10;13:RP101578. doi: 10.7554/eLife.101578 (PMC12422734; doi:10.7554/eLife.101578)

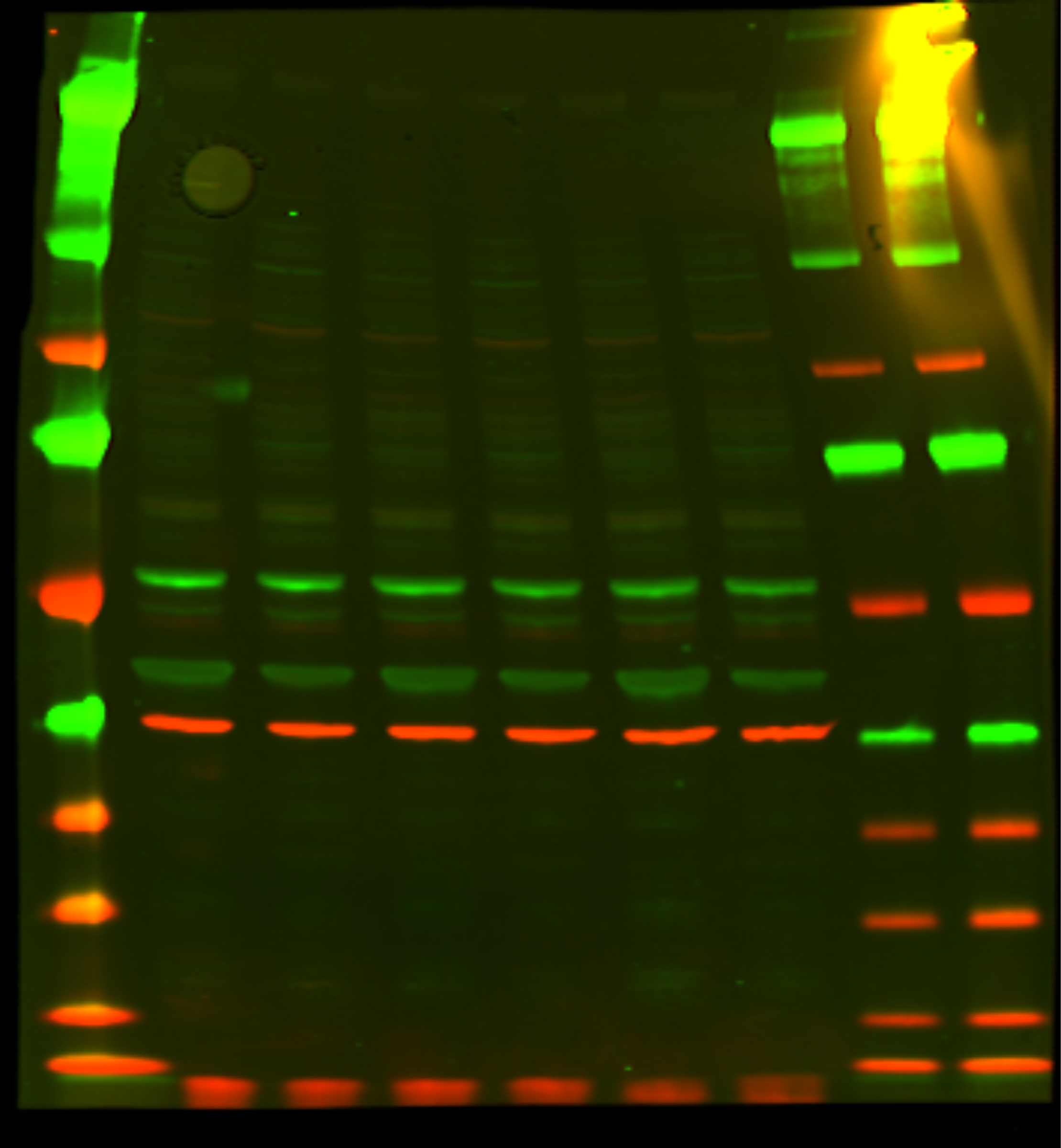

Supplement: Figure 5—source data 1. [file elife-101578-fig5-data1.zip › raw/CCN1_Triplicates_17OCTOBER2024.tif]

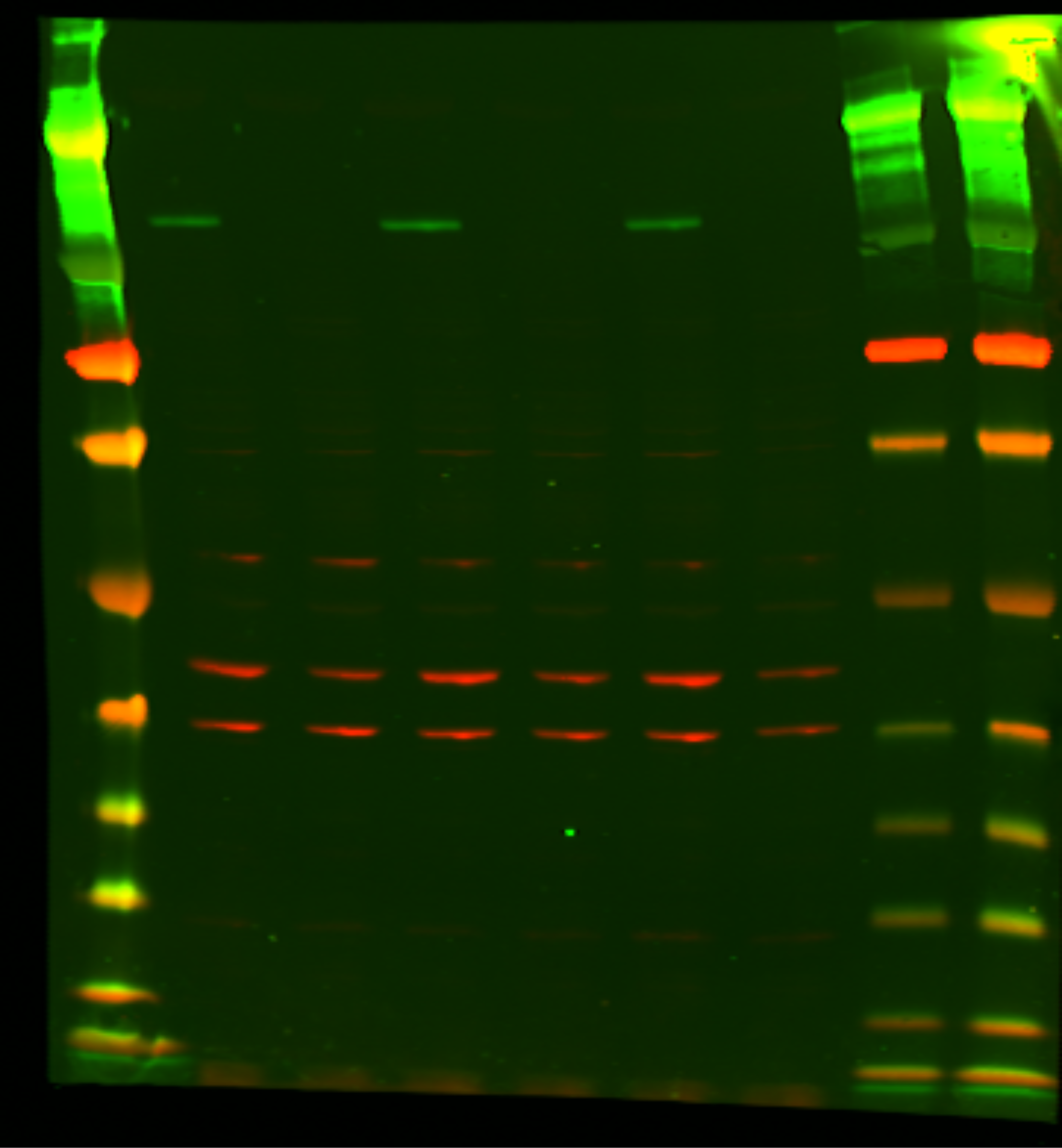

Supplement: Figure 5—source data 1. [file elife-101578-fig5-data1.zip › raw/THBS1_Triplicate_16OCTOBER2024.tif]

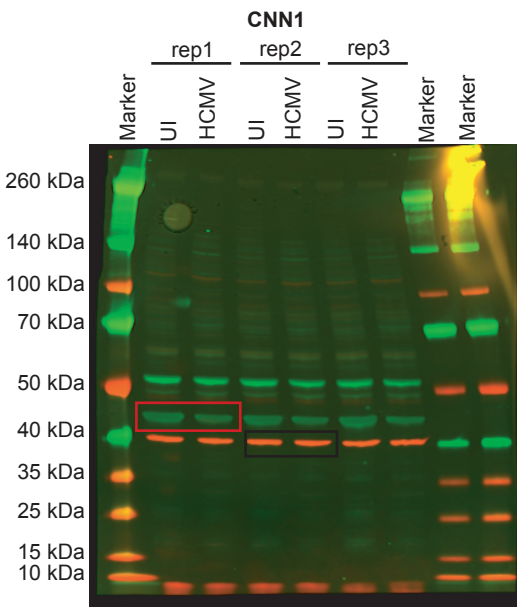

GAPDH

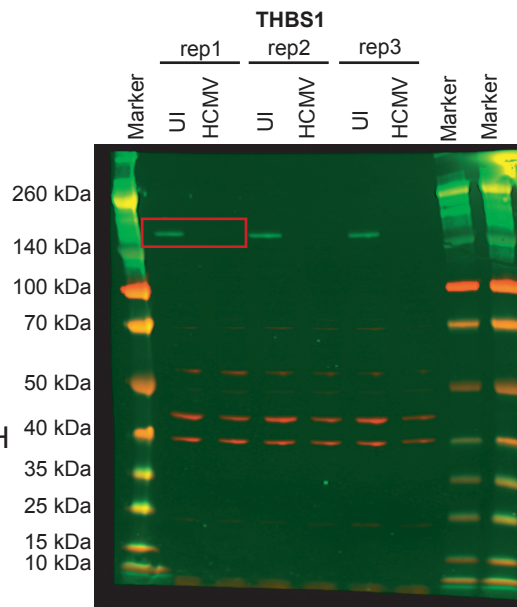

Supplement: Figure 5—source data 2. [file elife-101578-fig5-data2.zip › raw_with_labels/Figure 5_labeled.pdf]

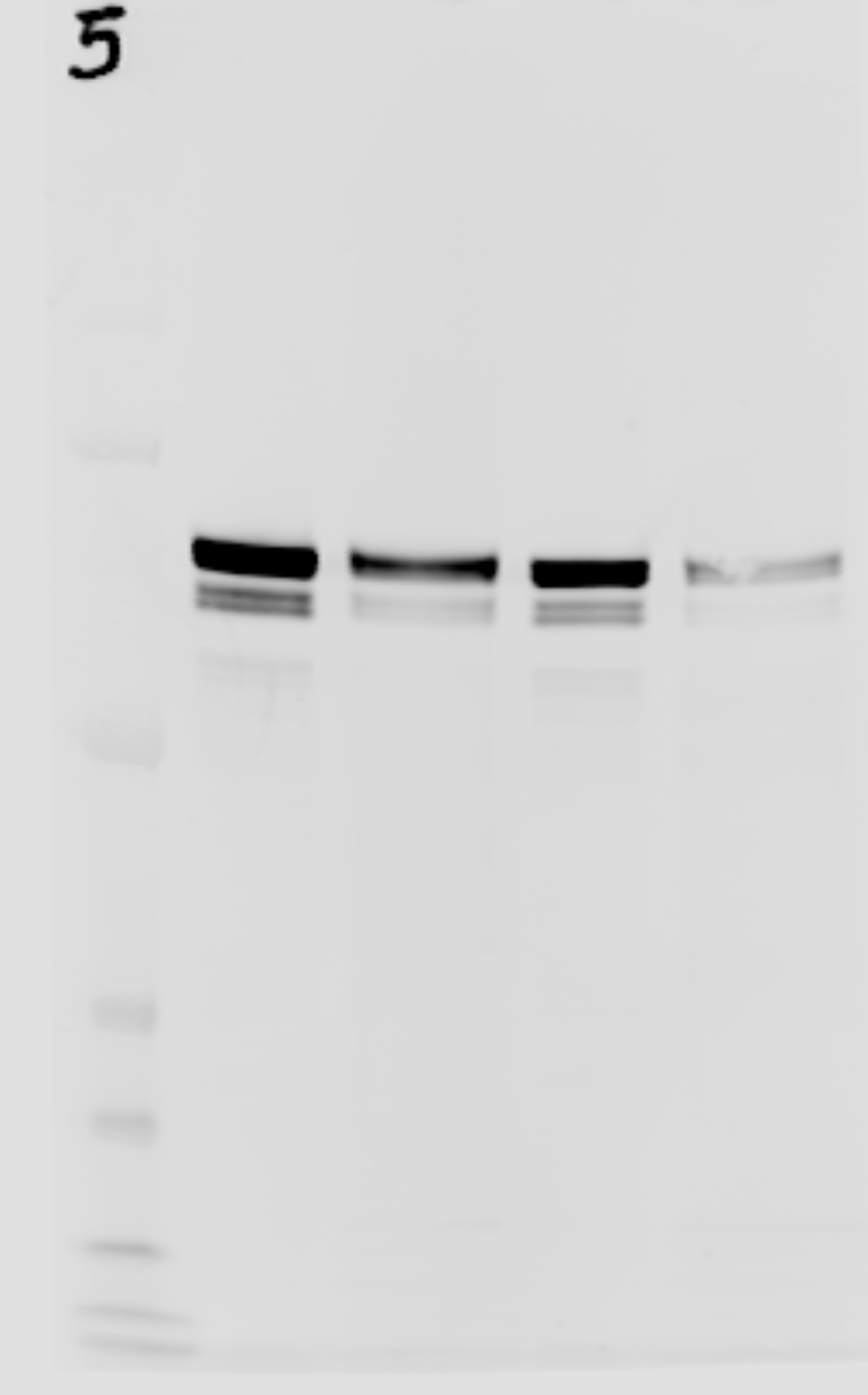

Supplement: Figure 6—source data 1. [file elife-101578-fig6-data1.zip › raw/YAP1_Cext_Next_20APR2023.png]

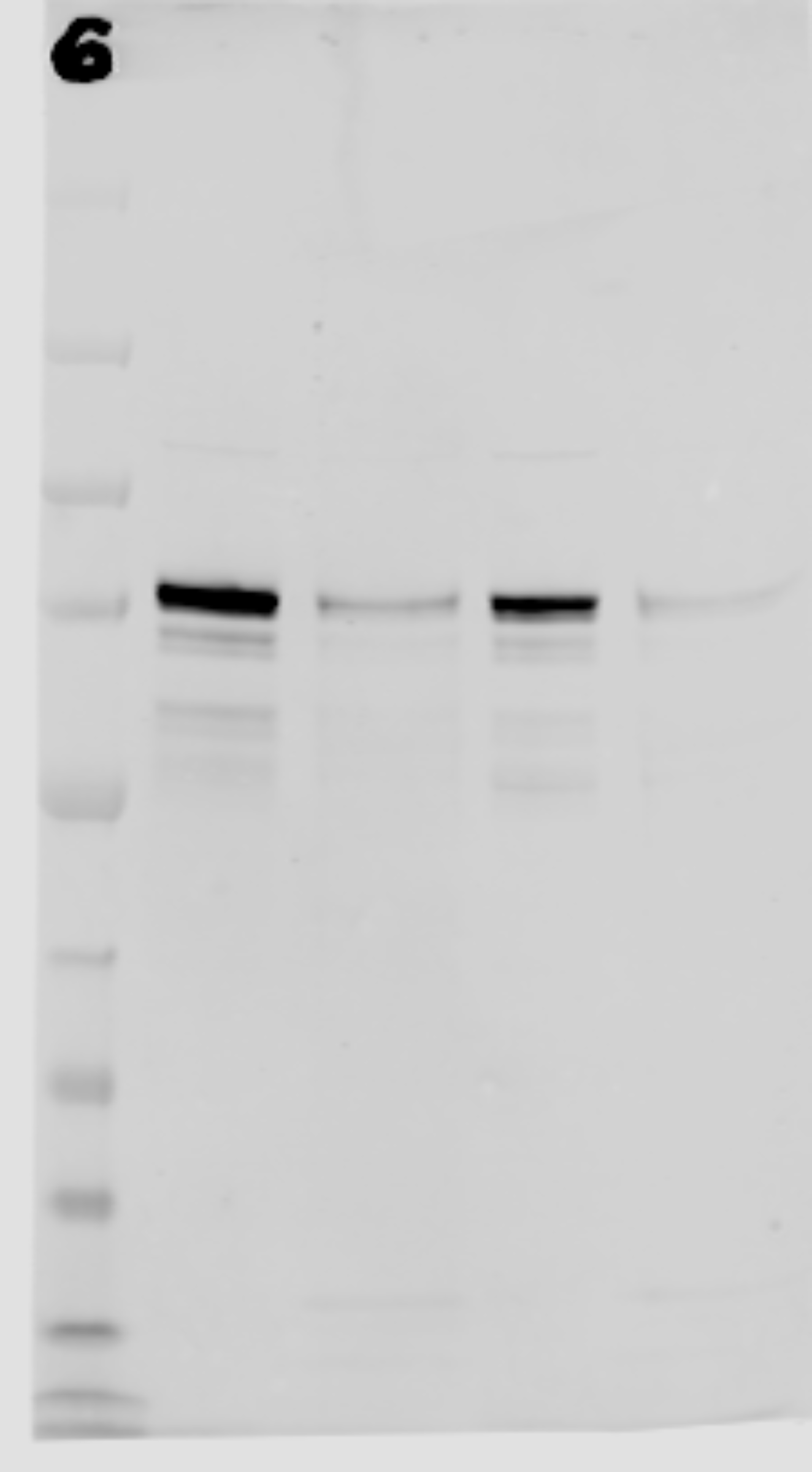

Supplement: Figure 6—source data 1. [file elife-101578-fig6-data1.zip › raw/pYAP1_Cext_Next_20APR2023.png]

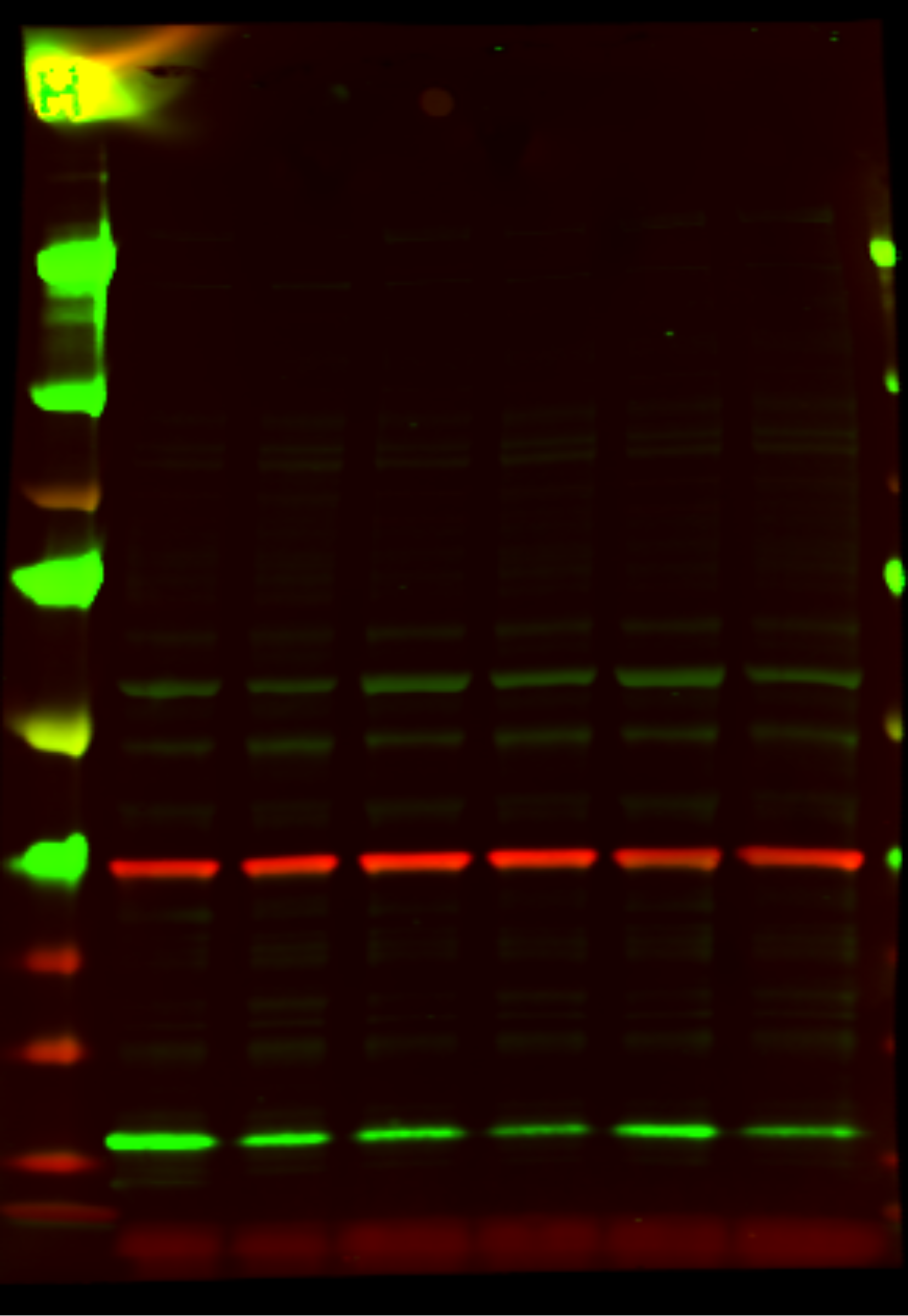

Supplement: Figure 6—source data 1. [file elife-101578-fig6-data1.zip › raw/H3K27ac_Pub_WB_Triplicates_21SEPT2023.tif]

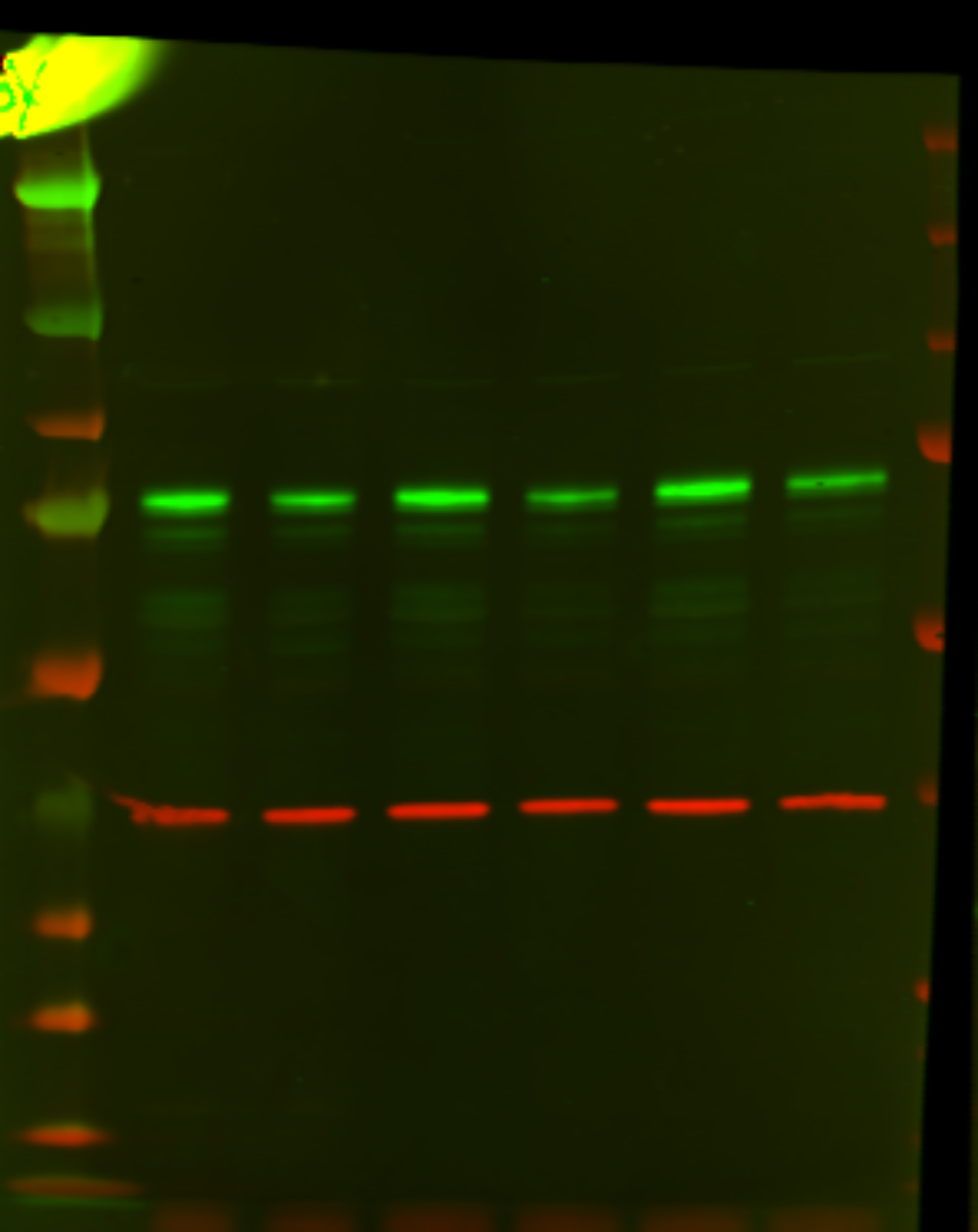

Supplement: Figure 6—source data 1. [file elife-101578-fig6-data1.zip › raw/pYAP1_Pub_WB_Triplicates_21SEPT2023.tif]

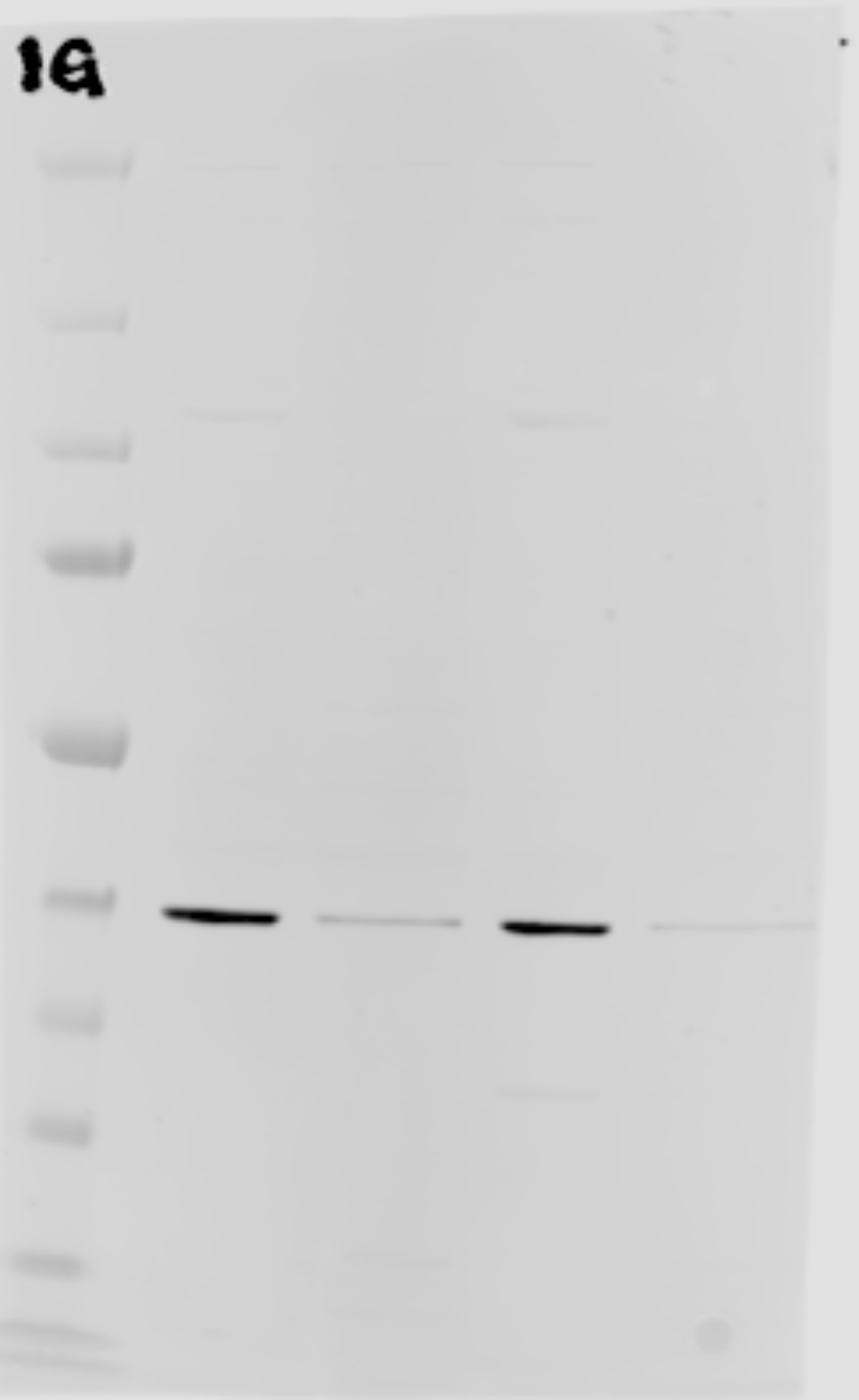

Supplement: Figure 6—source data 1. [file elife-101578-fig6-data1.zip › raw/GAPDH_C.ext_N.ext_27MAY2023.tif]

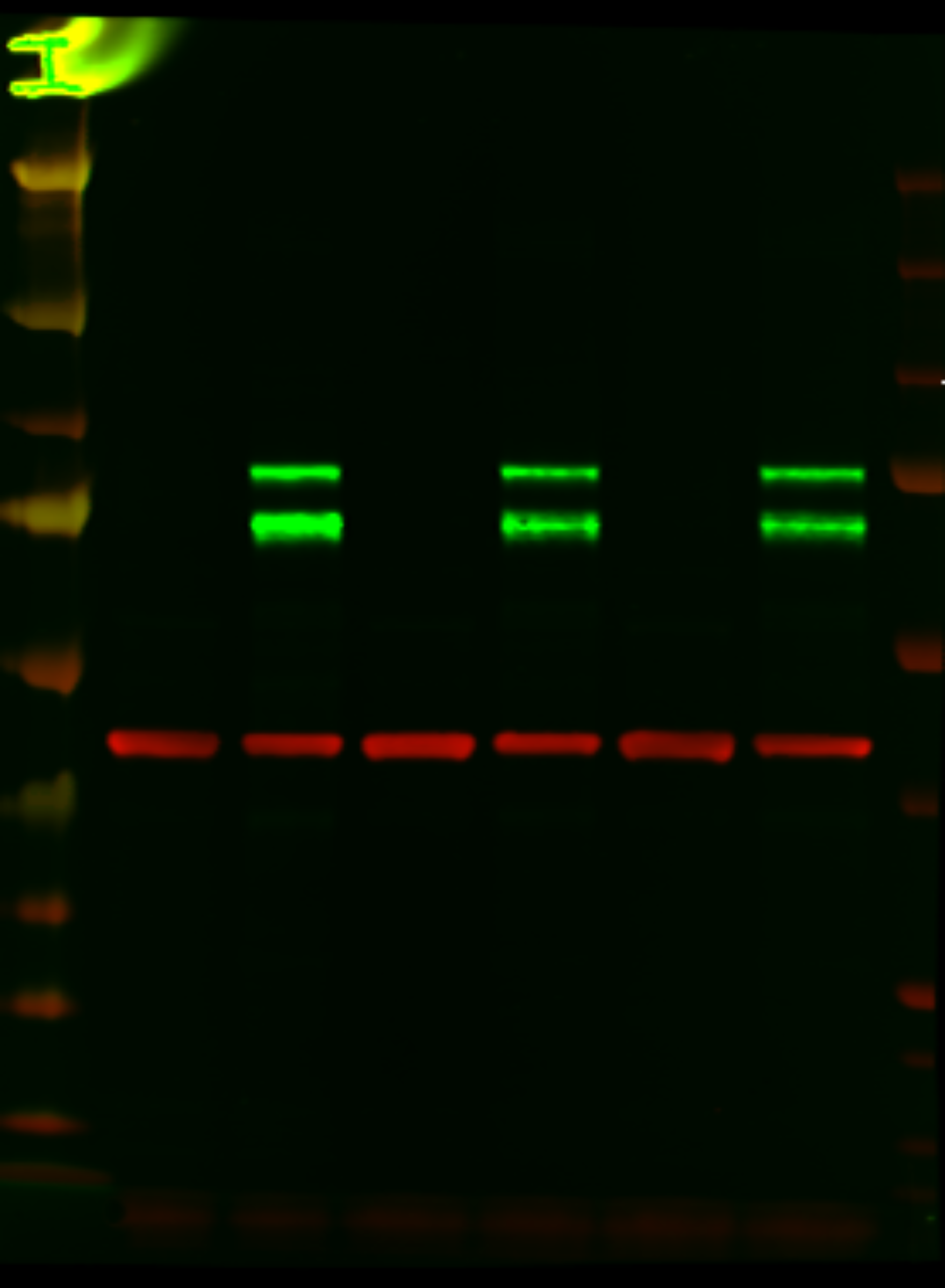

Supplement: Figure 6—source data 1. [file elife-101578-fig6-data1.zip › raw/IE1-2_Pub_WB_Triplicates_21SEPT2023.tif]

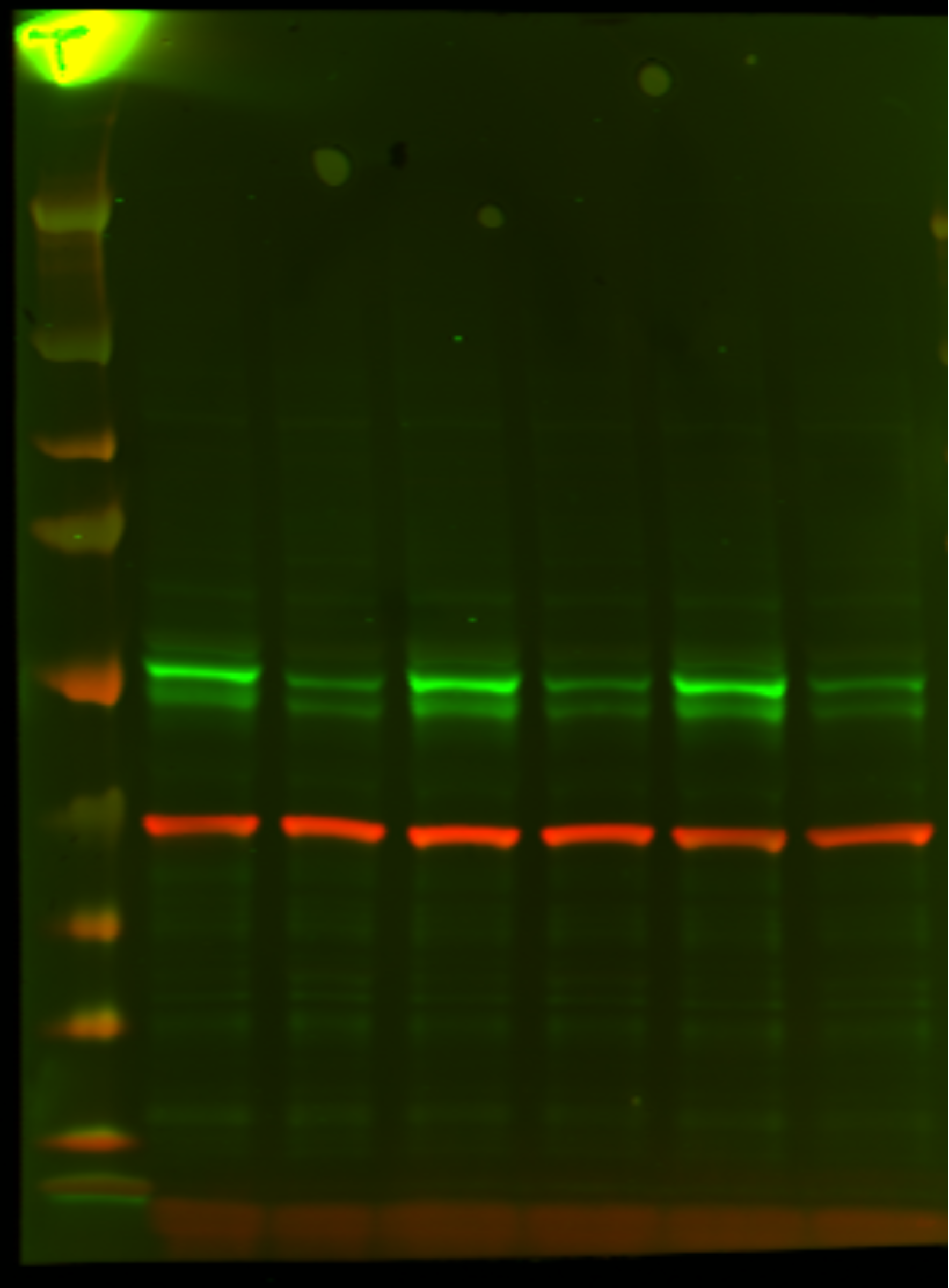

Supplement: Figure 6—source data 1. [file elife-101578-fig6-data1.zip › raw/TEAD1_Pub_WB_Triplicates_21SEPT2023.tif]

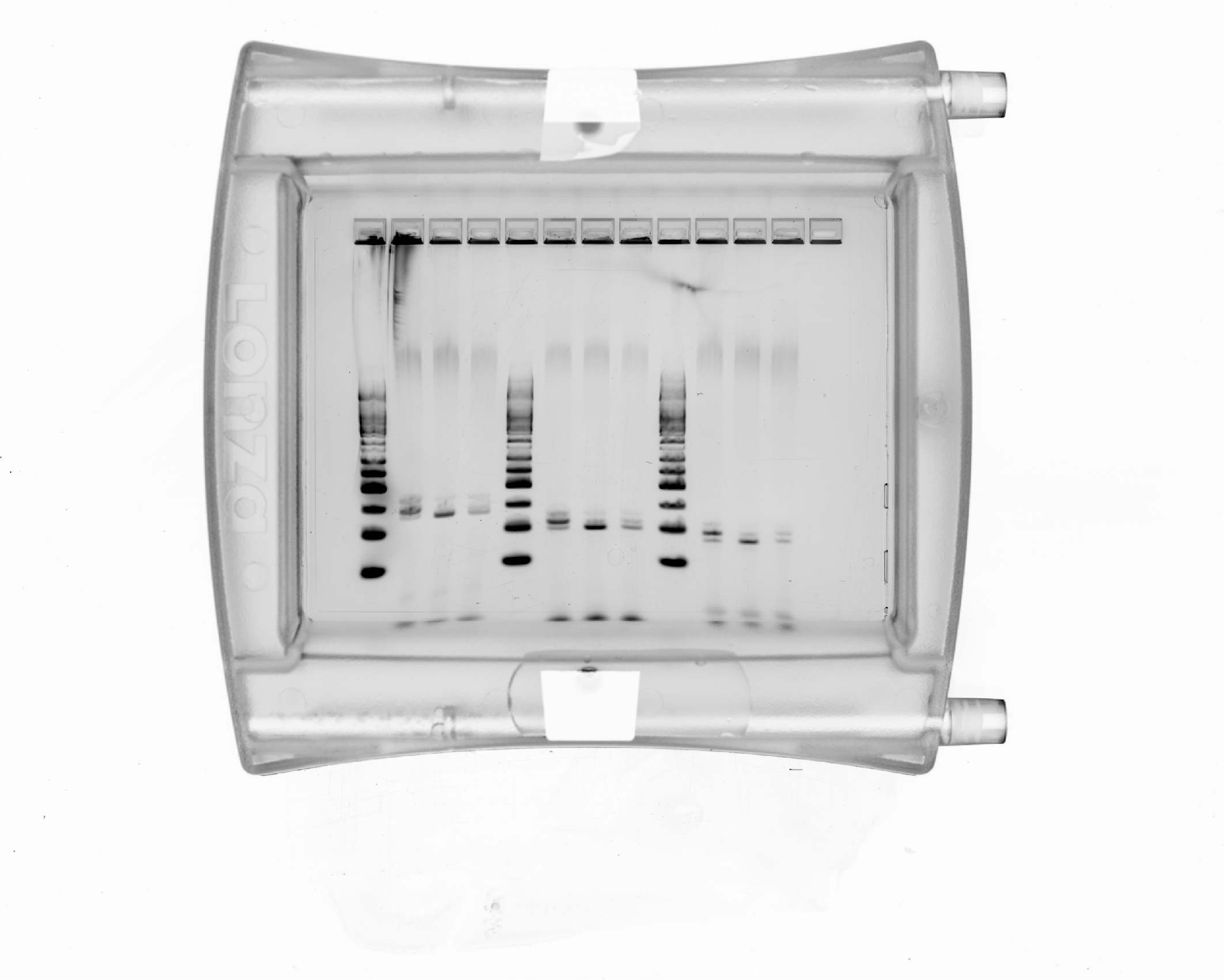

Supplement: Figure 6—source data 1. [file elife-101578-fig6-data1.zip › raw/lab 2023-03-16 22h35m29s(SYBR Gold).jpg]

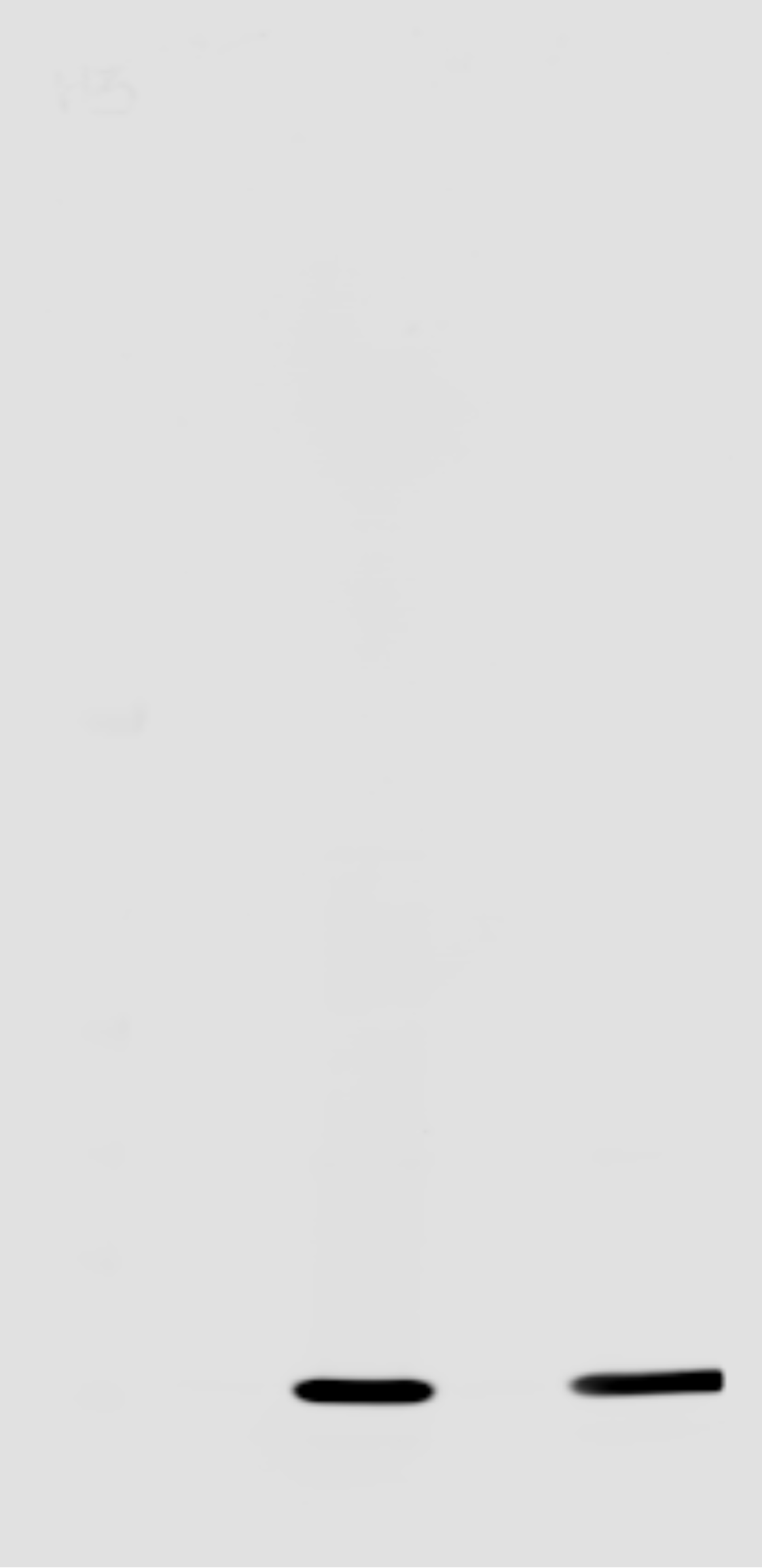

Supplement: Figure 6—source data 1. [file elife-101578-fig6-data1.zip › raw/Total Histone3_HFF_HCMV_Cyto_Nuc_06SEPT2023.png]

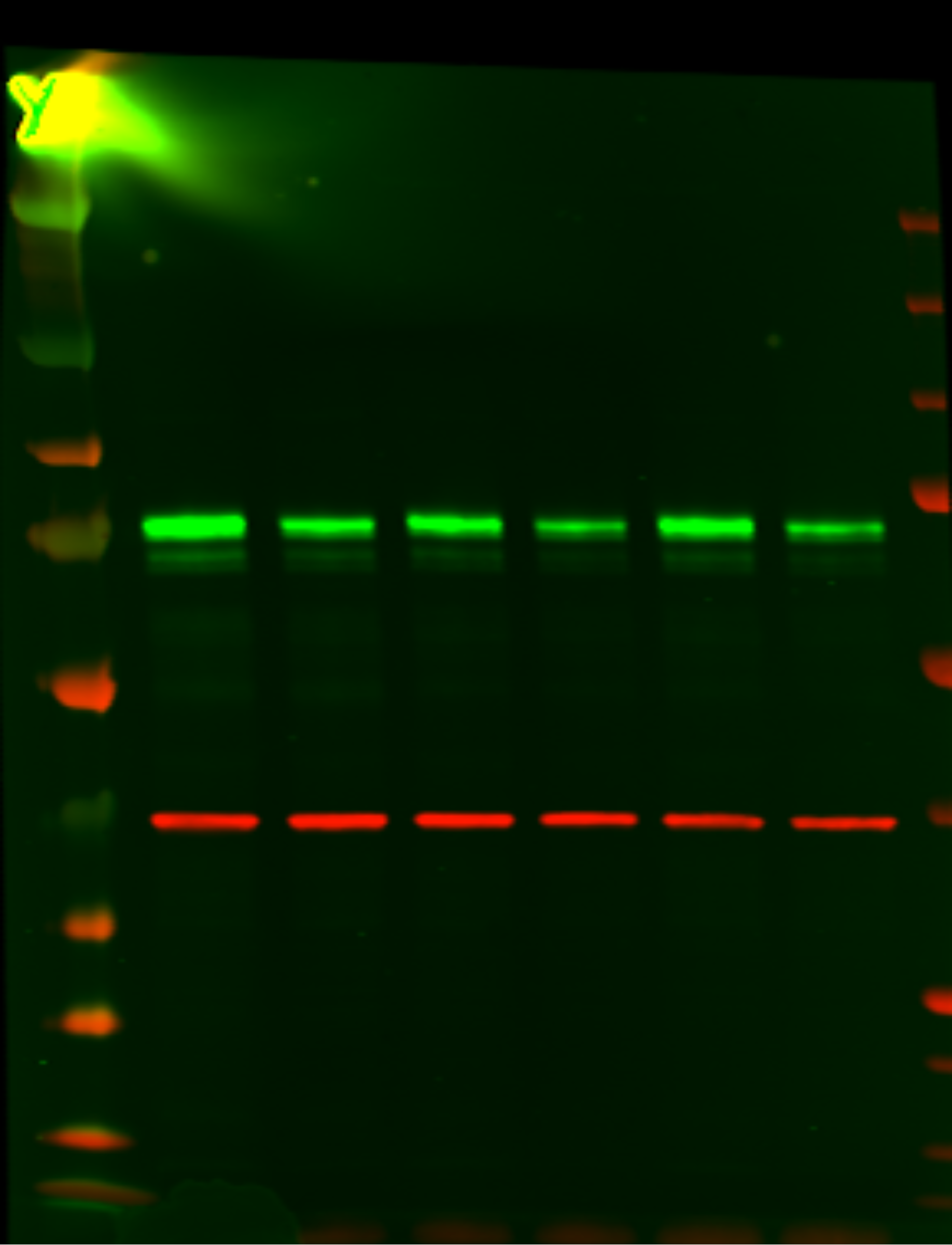

Supplement: Figure 6—source data 1. [file elife-101578-fig6-data1.zip › raw/YAP1_Pub_WB_Triplicates_21SEPT2023.tif]

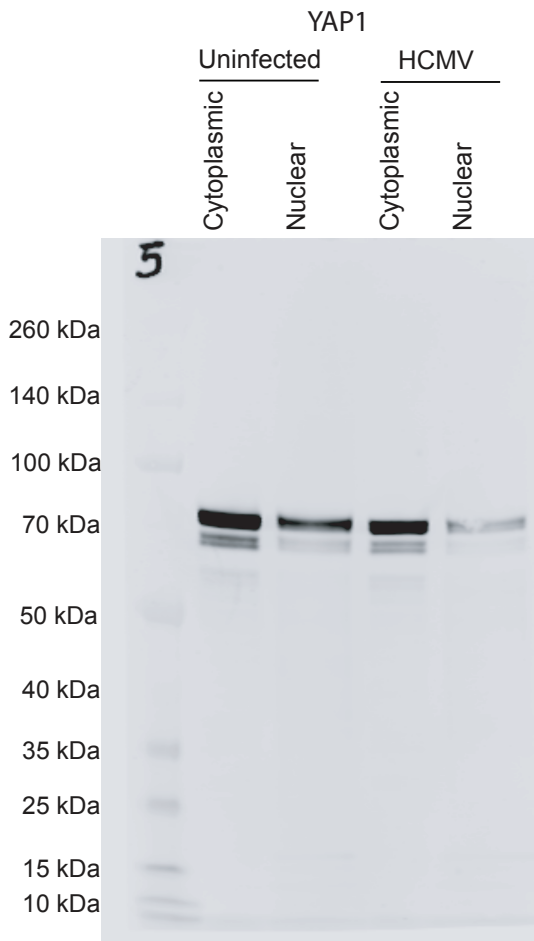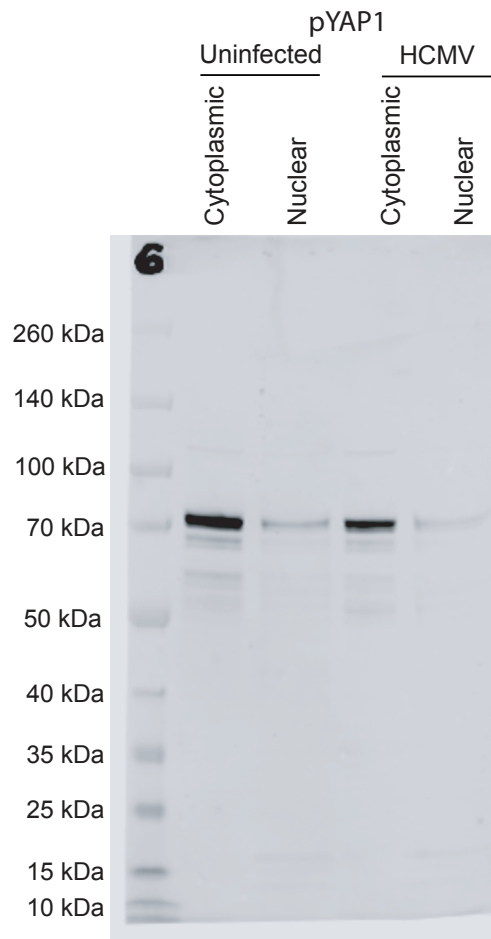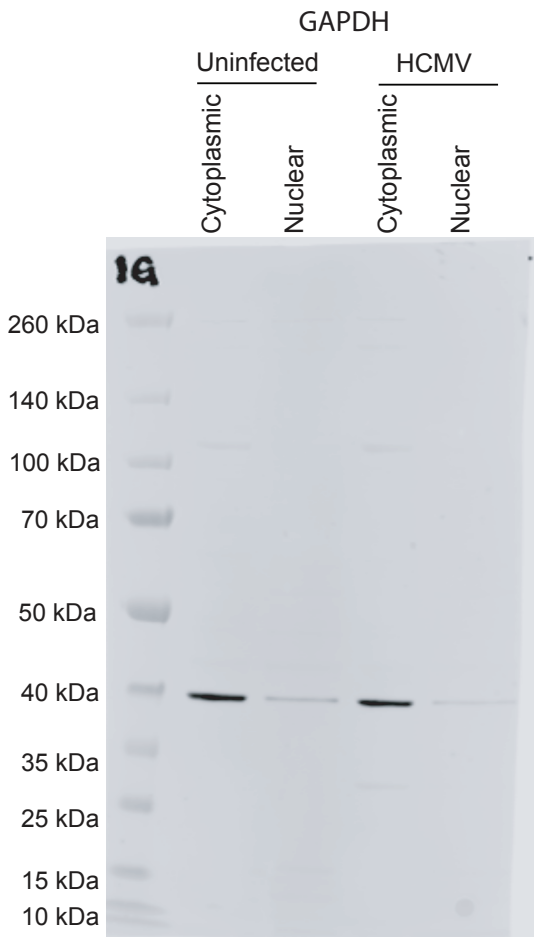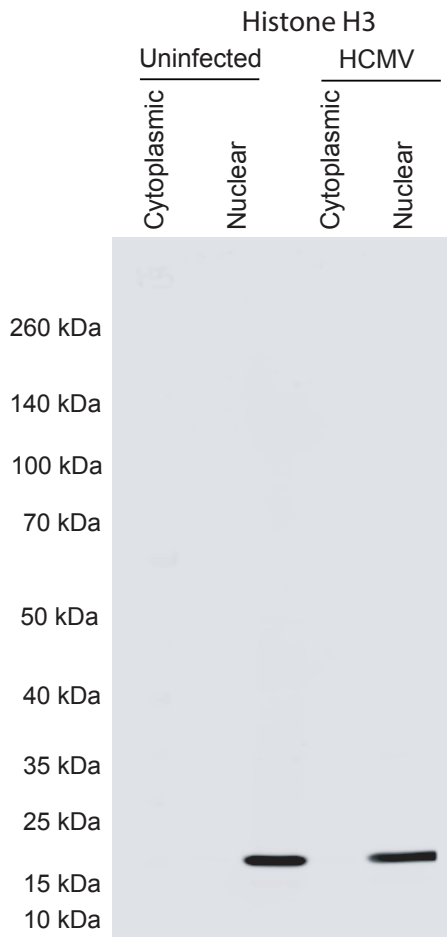

Supplement: Figure 6—source data 2. [file elife-101578-fig6-data2.zip › raw_labeled/Figure6B_labeled.pdf]

**Figure 6-figure supplement 1: Western blots of uninfected and HCMV infected fibroblasts**

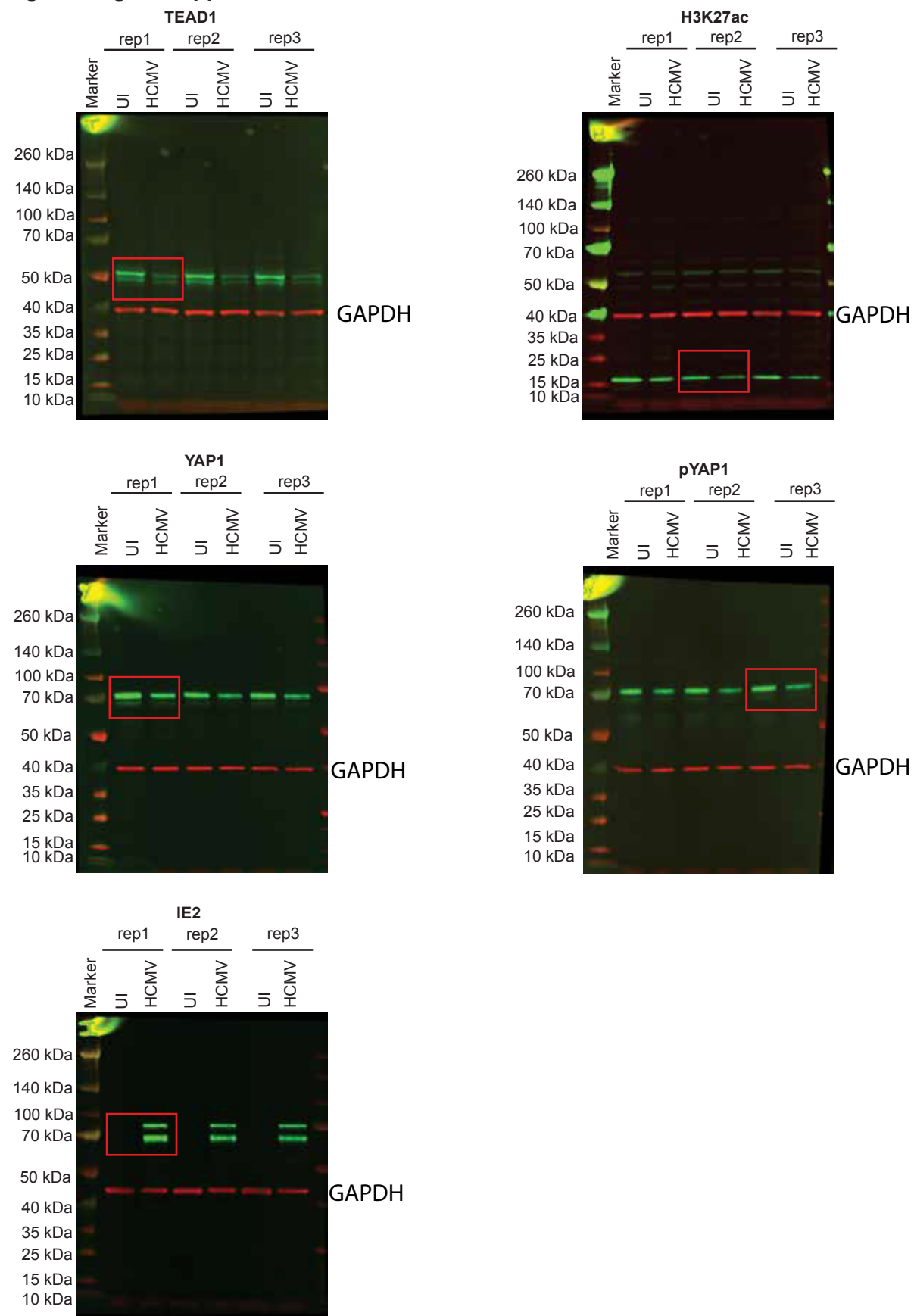

Supplement: Figure 6—source data 2. [file elife-101578-fig6-data2.zip › raw_labeled/Figure 6A_labeled.pdf]

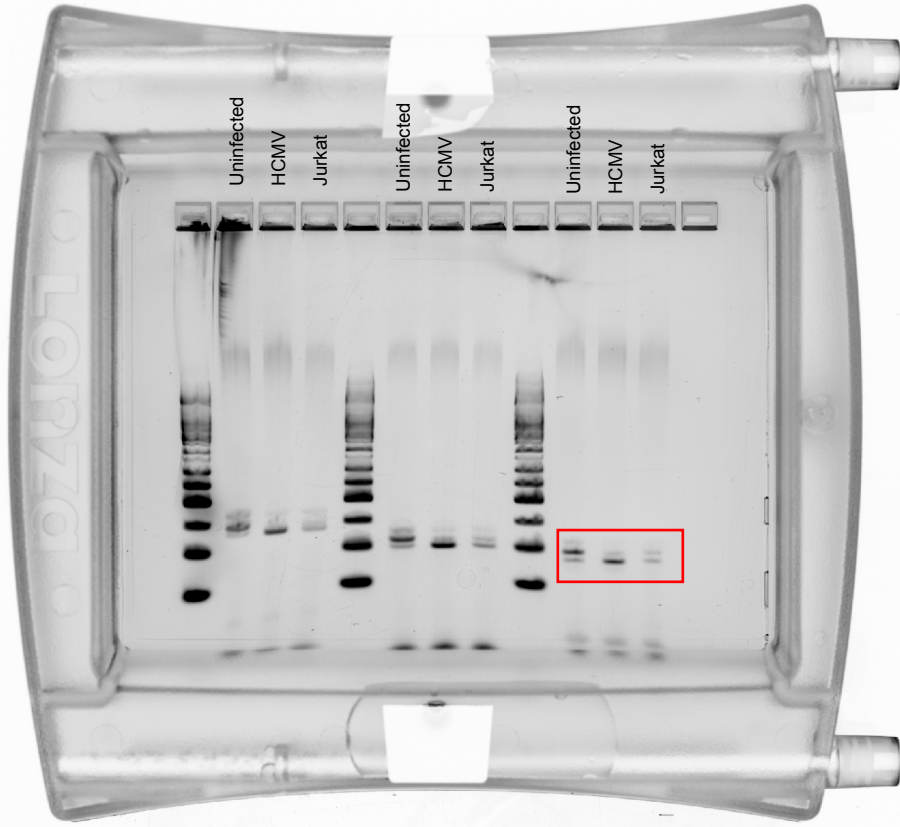

Supplement: Figure 6—source data 2. [file elife-101578-fig6-data2.zip › raw_labeled/figure6C_labeled.pdf]
