# Supplementary material for: Human cytomegalovirus infection coopts chromatin organization to diminish TEAD1 transcription factor activity
Source: eLife. 2025 Sep 10;13:RP101578. doi: 10.7554/eLife.101578 (PMC12422734; doi:10.7554/eLife.101578)
Supplement: MDAR checklist [file elife-101578-mdarchecklist1.docx]

**Materials Design Analysis Reporting (MDAR)**

**Checklist for Authors**

The [MDAR framework](https://osf.io/xfpn4/) establishes a minimum set of requirements in transparent reporting mainly applicable to studies in the life sciences.

*eLife* asks authors to **provide detailed information within their article** to facilitate the interpretation and replication of their work. Authors can also upload supporting materials to comply with relevant reporting guidelines for health-related research (see [EQUATOR Network](http://www.equator-network.org/%20)), life science research (see the [BioSharing Information Resource](http://biosharing.org/)), or animal research (see the [ARRIVE Guidelines](http://www.plosbiology.org/article/info:doi/10.1371/journal.pbio.1000412) and the [STRANGE Framework](https://doi.org/10.1038/d41586-020-01751-5); for details, see *eLife*’s [Journal Policies](https://reviewer.elifesciences.org/author-guide/journal-policies)). Where applicable, authors should refer to any relevant reporting standards materials in this form.

For all that apply, please note **where in the article** the information is provided. Please note that we also collect information about data availability and ethics in the submission form.

**Materials:**

| **Newly created materials** | **Indicate where provided: section/figure legend** | **N/A** |
| --- | --- | --- |
| The manuscript includes a dedicated "materials availability statement" providing transparent disclosure about availability of newly created materials including details on how materials can be accessed and describing any restrictions on access. |  | NA |
|  |  |  |
| **Antibodies** | **Indicate where provided: section/figure legend** | **N/A** |
| For commercial reagents, provide supplier name, catalogue number and [RRID](https://scicrunch.org/resources), if available. | These are found in the Chromatin immunoprecipitation sequencing (ChIP-seq) section and Assessment of YAP1 localization section of Materials and Methods |  |
|  |  |  |
| **DNA and RNA sequences** | **Indicate where provided: section/figure legend** | **N/A** |
| Short novel DNA or RNA including primers, probes: Sequences should be included or deposited in a public repository. | Primers are shown in Validation of TEAD1 splicing (RT-PCR) section of Materials and Methods |  |
|  |  |  |
| **Cell materials** | **Indicate where provided: section/figure legend** | **N/A** |
| Cell lines: Provide species information, strain. Provide accession number in repository OR supplier name, catalog number, clone number, OR RRID. | the statement “Experiments were performed in human foreskin fibroblasts (HS68 cells). ATAC-seq experiments were also performed in human retinal epithelial cells (ARPE-19 cells). HS68 and ARPE-19 cell lines were obtained from ATCC.” is included in the “Experimental design, cell culture, and viral infections” section of Materials and Methods |  |
| Primary cultures: Provide species, strain, sex of origin, genetic modification status. |  | NA |
|  |  |  |
| **Experimental animals** | **Indicate where provided: section/figure legend** | **N/A** |
| Laboratory animals or Model organisms: Provide species, strain, sex, age, genetic modification status. Provide accession number in repository OR supplier name, catalog number, clone number, OR RRID. |  | NA |
| Animal observed in or captured from the field: Provide species, sex, and age where possible. |  | NA |
|  |  |  |
| **Plants and microbes** | **Indicate where provided: section/figure legend** | **N/A** |
| Plants: provide species and strain, ecotype and cultivar where relevant, unique accession number if available, and source (including location for collected wild specimens). |  | NA |
| Microbes: provide species and strain, unique accession number if available, and source. |  | NA |
|  |  |  |
| **Human research participants** | **Indicate where provided: section/figure legend) or state if these demographics were not collected** | **N/A** |
| If collected and within the bounds of privacy constraints report on age, sex, gender and ethnicity for all study participants. |  | NA |

**Design:**

| **Study protocol** | **Indicate where provided: section/figure legend** | **N/A** |
| --- | --- | --- |
| If the study protocol has been pre-registered, provide DOI. For clinical trials, provide the trial registration number OR cite DOI. |  | NA |
|  |  |  |
| **Laboratory protocol** | **Indicate where provided: section/figure legend** | **N/A** |
| Provide DOI OR other citation details if detailed step-by-step protocols are available. |  | NA |
|  |  |  |
| **Experimental study design (statistics details) *** | | |
| **For in vivo studies: State whether and how the following have been done** | **Indicate where provided: section/figure legend. If it could have been done, but was not, write “not done”** | **N/A** |
| Sample size determination |  | NA |
| Randomisation |  | NA |
| Blinding |  | NA |
| Inclusion/exclusion criteria |  | NA |
|  |  |  |
| **Sample definition and in-laboratory replication** | **Indicate where provided: section/figure legend** | **N/A** |
| State number of times the experiment was replicated in the laboratory. | “ATAC-seq experiments were performed in duplicate in uninfected conditions or 48 hours post infection (hpi) (Figure 1B).” in the Results section  “ChIP-seq experiments for TEAD1, CTCF, and H3K27ac were performed in duplicate in uninfected and HCMV infected conditions (48 hours post infection).” in the Results section,  “Uninfected and HCMV infected cells were harvested, in triplicates” in “Assessment of protein expression levels (Western blots)” in Materials and Methods |  |
| Define whether data describe technical or biological replicates. | All are biological replicates |  |
|  |  |  |
| **Ethics** | **Indicate where provided: section/submission form** | **N/A** |
| Studies involving human participants: State details of authority granting ethics approval (IRB or equivalent committee(s), provide reference number for approval. |  | NA |
| Studies involving experimental animals: State details of authority granting ethics approval (IRB or equivalent committee(s), provide reference number for approval. |  | NA |
| Studies involving specimen and field samples: State if relevant permits obtained, provide details of authority approving study; if none were required, explain why. |  | NA |
|  |  |  |
| **Dual Use Research of Concern (DURC)** | **Indicate where provided: section/submission form** | **N/A** |
| If study is subject to dual use research of concern regulations, state the authority granting approval and reference number for the regulatory approval. |  | NA |

**Analysis:**

| **Attrition** | **Indicate where provided: section/figure legend** | **N/A** |
| --- | --- | --- |
| Describe whether exclusion criteria were pre-established. Report if sample or data points were omitted from analysis. If yes, report if this was due to attrition or intentional exclusion and provide justification. | Exclusion criteria were pre-established in that events that were not detected in all replicates in each biological group were excluded from the differential analyses" in the materials and methods section. |  |
|  |  |  |
| **Statistics** | **Indicate where provided: section/figure legend** | **N/A** |
| Describe statistical tests used and justify choice of tests. | Materials and Methods:  “Finally, differential gene expression analysis was performed using DESeq2 (version 1.30.1) (Love et al., 2014). Genes were considered differentially expressed if they had a 2-fold change and an adjusted p-value threshold of less than 0.01. Pathway enrichment analyses for the differentially expressed genes were performed using Enrichr (Chen et al., 2013; Kuleshov et al., 2016). An adjusted p-value threshold of 0.05 was used to generate the pathway enrichment figure (Figure 5B).”  “Exon-exon and exon-intron spanning reads were identified with the software AltAnalyze (version 2.1.4) using the Ensembl-72 human database, along with splicing event calculation and annotation with the MultiPath-PSI algorithm (see: http://altanalyze.readthedocs.io/en/latest/Algorithms for algorithm details and benchmarking).” … “Significant splicing changes were defined as splicing events with a change in percent spliced in (PSI) value between the two groups > 10% (ΔPSI > \| 0.1\|), with p-value <0.01.”  “Differential chromatin accessibility analysis was performed using DiffBind 3.6.5 (Rory Stark, 2017; Ross-Innes et al., 2012) in R 4.2.1 (Team, 2023). The “conservative overlap” peak sets generated from the individual replicates by the ENCODE ATAC-seq pipeline were used for differential analysis. Peaks were considered to be differentially accessible if the FDR was less than 0.01 and the fold change was 2-fold or greater. A modified version of HOMER (Heinz et al., 2010) using a log base 2 likelihood scoring system was used to calculate motif enrichment statistics for a large library of human position weight matrix (PWM) binding site models contained in build 2.0 of the CisBP database (Weirauch et al., 2014). DeepTools (v2.0.0) (Ramirez et al., 2016) was used to generate heatmaps of signal tracks across differentially accessible chromatin for each set of comparisons.”  “ChIP-seq data were processed and aligned to the hg19 genome using the ENCODE ChIP-seq pipeline (V2.0.0) (Consortium, 2012; Hitz et al., 2023; Lee et al., 2021b; Luo et al., 2020). Peaks were called within the pipeline using MACS2 (Zhang et al., 2008). Differential peak analysis was performed using DiffBind 3.6.5 (Rory Stark, 2017; Ross-Innes et al., 2012) in R 4.2.1 (Team, 2023). The “conservative overlap” peak sets generated from the individual replicates by the ENCODE ChIP-seq pipeline were used for differential analysis. Peaks were considered differentially enriched if the FDR was less than 0.01 and the fold change was 2-fold or greater.”  “This created a list of disease risk loci, along with the corresponding genetic variants within the LD block. This list of variants was then used for RELI analyses as previously published (Harley et al., 2018). Phenotypes were considered “TEAD1 loss specific” if: (1) they had 3 or more overlaps with TEAD1 binding event losses; (2) this overlap was significant according to RELI (corrected p<0.01); (3) this overlap was not significant for unchanged TEAD1 peaks (p>0.01), and (4) TEAD1 binding loss events were at least 2-fold enriched according to RELI.” |  |
|  |  |  |
| **Data availability** | **Indicate where provided: section/submission form** | **N/A** |
| For newly created and reused datasets, the manuscript includes a data availability statement that provides details for access (or notes restrictions on access). | This is covered in the “data access” section. |  |
| When newly created datasets are publicly available, provide accession number in repository OR DOI and licensing details where available. | Datasets are deposited in GEO and a UCSC Genome Browser session listed in the data access statement |  |
| If reused data is publicly available provide accession number in repository OR DOI, OR URL, OR citation. | Found in supplementary file 1 |  |
|  |  |  |
| **Code availability** | **Indicate where provided: section/figure legend** | **N/A** |
| For any computer code/software/mathematical algorithms essential for replicating the main findings of the study, whether newly generated or re-used, the manuscript includes a data availability statement that provides details for access or notes restrictions. | All code is specified, particularly in what parameters were used. For example, “MboI restriction sites and default parameters were used (in HiC-Pro) to align the reads to the hg19 genome.”  “The parameters for FitHiChIP were the following: interaction type of “peak to all”; bin size of 10 kb; lower loop distance of 20 kb; upper loop distance of 20 mb; background model of loose; bias correction of coverage bias regression; merge filtering enabled; and FDR of 0.01”  “The parameters used for multiBamSummary were --binSize 10000 and --distanceBetweenBins 0. The parameters used for plotCorrelation were --corMethod pearson, --skipZeros, and -- removeOutliers.”  “Signal tracks (in bigWig format) were created using the bamCoverage program from the deepTools software package (Ramirez et al., 2016) with the parameters --normalizeUsing BPM and --binSize 10. For the RNA-seq signal tracks, the command was run twice in order to maintain strandedness. For the forward strand track, the parameter --filterRNAstrand forward was included. For the reverse strand track, the parameters --filterRNAstrand reverse and --scaleFactor -1 were included. The .hic files for the HiChIP data were created using the hicpro2juicebox.sh script from HiC-Pro (version 2.11.4) (Servant et al., 2015). Juicer (version 1.22.01) (Durand et al., 2016) was used for KR normalization.”  “The .hic files for the HiChIP data were created using the hicpro2juicebox.sh script from HiC-Pro (version 2.11.4) (Servant et al., 2015)” |  |
| Where newly generated code is publicly available, provide accession number in repository, OR DOI OR URL and licensing details where available. State any restrictions on code availability or accessibility. |  | NA |
| If reused code is publicly available provide accession number in repository OR DOI OR URL, OR citation. |  | NA |

**Reporting:**

The MDAR framework recommends adoption of discipline-specific guidelines, established and endorsed through community initiatives.

| **Adherence to community standards** | **Indicate where provided: section/figure legend** | **N/A** |
| --- | --- | --- |
| State if relevant guidelines (e.g., ICMJE, MIBBI, ARRIVE, STRANGE) have been followed, and whether a checklist (e.g., CONSORT, PRISMA, ARRIVE) is provided with the manuscript. |  | NA |

* We provide the following guidance regarding transparent reporting and statistics; we also refer authors to [Ten common statistical mistakes to watch out for when writing or reviewing a manuscript](https://doi.org/10.7554/eLife.48175).

**Sample-size estimation**

- You should state whether an appropriate sample size was computed when the study was being designed
- You should state the statistical method of sample size computation and any required assumptions
- If no explicit power analysis was used, you should describe how you decided what sample (replicate) size (number) to use

**Replicates**

- You should report how often each experiment was performed
- You should include a definition of biological versus technical replication
- The data obtained should be provided and sufficient information should be provided to indicate the number of independent biological and/or technical replicates
- If you encountered any outliers, you should describe how these were handled
- Criteria for exclusion/inclusion of data should be clearly stated
- High-throughput sequence data should be uploaded before submission, with a private link for reviewers provided (these are available from both GEO and ArrayExpress)

**Statistical reporting**

- Statistical analysis methods should be described and justified
- Raw data should be presented in figures whenever informative to do so (typically when N per group is less than 10)
- For each experiment, you should identify the statistical tests used, exact values of N, definitions of center, methods of multiple test correction, and dispersion and precision measures (e.g., mean, median, SD, SEM, confidence intervals; and, for the major substantive results, a measure of effect size (e.g., Pearson's r, Cohen's d)
- Report exact p-values wherever possible alongside the summary statistics and 95% confidence intervals. These should be reported for all key questions and not only when the p-value is less than 0.05.

**Group allocation**

- Indicate how samples were allocated into experimental groups (in the case of clinical studies, please specify allocation to treatment method); if randomization was used, please also state if restricted randomization was applied
- Indicate if masking was used during group allocation, data collection and/or data analysis
